# The Prophylactic Effect of Pinocembrin Against Doxorubicin-Induced Cardiotoxicity in an *In Vitro* H9c2 Cell Model

**DOI:** 10.3389/fphar.2020.01172

**Published:** 2020-08-05

**Authors:** Nonhlakanipho F. Sangweni, Malebogo Moremane, Sylvia Riedel, Derick van Vuuren, Barbara Huisamen, Lawrence Mabasa, Reenen Barry, Rabia Johnson

**Affiliations:** ^1^ Biomedical Research and Innovation Platform (BRIP), South African Medical Research Council, Tygerberg, South Africa; ^2^ Division of Medical Physiology, Faculty of Health Sciences, Stellenbosch University, Tygerberg, South Africa; ^3^ Research and Development Department, Biopharm, Hamilton, New Zealand

**Keywords:** doxorubicin, cardiotoxicity, pinocembrin, antioxidants, mitochondrial bioenergetics, apoptosis

## Abstract

**Background:**

The clinical use of Doxorubicin (Dox) is significantly limited by its dose-dependent cardiotoxic side effect. Accumulative evidence suggests that the use of flavonoids, such as the antioxidative Pinocembrin (Pin), could be effective in the prevention of Dox-induced cardiotoxicity. Accordingly, we investigated the ability of pinocembrin (Pin) to attenuate Dox-induced cardiotoxicity in an in vitro H9c2 cardiomyoblast model.

**Methodology:**

The cardioprotective potential of Pin was established in H9c2 cells. Here, cells were treated with Dox (2μM), Dox (2μM) + Pin (1μM), and Dox (2μM) + Dexrazoxane (20μM) for 6 days. Thereafter, the safe co-administration of Pin with Dox, in a cancer environment, was investigated in MCF-7 breast cancer cells subjected to the same experimental conditions. Untreated cells served as the control. Subsequently, Pin’s ability to attenuate Dox-mediated oxidative stress, impaired mitochondrial bioenergetics and potential, as well as aggravated apoptosis was quantified using biochemical assays.

**Results:**

The results demonstrated that co-treatment with Pin mitigates Dox-induced oxidative stress by alleviating the antioxidant enzyme activity of the H9c2 cells. Pin further reduced the rate of apoptosis and necrosis inferred by Dox by improving mitochondrial bioenergetics. Interestingly, Pin did not decrease the efficacy of Dox but, rather increased the rate of apoptosis and necrosis in Dox-treated MCF-7 cells.

**Conclusion:**

The findings presented in this study showed, for the first time, that Pin attenuates Dox-induced cardiotoxicity without reducing its chemotherapeutic effect. We propose that additional studies, using in vivo models, should be conducted to further investigate Pin as a suitable candidate in the prevention of the cardiovascular dysfunction inferred by Dox administration.

## Introduction

Doxorubicin (Dox) is a highly potent chemotherapeutic drug that is actively used in the treatment of numerous malignancies like metastatic breast cancer, however, the clinically use of Dox is hindered by a dose-dependent cardiotoxic side effect (Koleini and Kardami, 2017; Zilinyi et al., 2018). This has been confirmed in various studies including studies conducted by Harake et al. (2012) and Zilinyi et al. (2018) who argued that while the use of Dox increased life expectancy, its use as a chemotherapeutic agent was limited.

While the molecular signaling pathways underlying the cardiotoxic side-effects of Dox remain obscure, several theories including mitochondrial dysfunction, increased reactive oxygen species (ROS) production and aggravated apoptosis have been proposed as plausible underlying mechanisms (Carvalho et al., 2009; McGowan et al., 2017). Singal and Kirshenbaum (1990) suggested that a possible strategy to prevent Dox-induced cardiotoxicity would be to target these pathways and introduce additional antioxidant therapy to Dox treatment. However, in the case of cancer cells they exhibit higher basal oxidative stress relative to normal cells and consequently take advantage of the upregulated antioxidant system to circumvent ROS-mediated tumor cell damage (Raninga et al., 2014; Bernardes et al., 2015; Thyagarajan and Sahu, 2018). Additionally, Yasueda and colleagues (2016) argued that while antioxidant therapies are capable of alleviating the adverse effects of chemotherapy, they could antagonize the antitumor effects by reducing oxidative damage. Thus, it is imperative to clarify whether or not newly developed therapeutic adjuncts interact with cancer therapy. Currently, the only FDA approved cardioprotective agent used to reduce the burden inferred by Dox on the myocardium is Dexrazoxane (Dex) (Vachhani et al., 2017). Initially, the use of Dex was surrounded by controversy possibly because of concerns of its potential impact on chemotherapy efficacy and the risk of secondary malignant neoplasms (Kosty et al., 2001; Levis et al., 2017; Tahover et al., 2017). However, a meta-analysis by van Dalen et al. (2011) reported that Dex provided a benefit on heart failure with a risk ratio of 0.29 and no deleterious effect on the overall survival. Nonetheless, on the basis of these findings, the European Medicines Agency has since restricted the use of Dex to adult patients and contraindicating its use in pediatric patients. This therefore, necessitates the need to investigate alternative treatment to use as adjuncts to current chemotherapeutic drugs (Ganatra et al., 2019).

Such a treatment could be Pinocembrin (Pin, 5,7-dihydroxyflavanone), which is an antioxidative flavanone (Lungkaphin et al., 2015; Ahmeda et al., 2017) that has been identified in propolis and several plants, such as *Galenia africana*, and is reported to have anti-cancer and cardioprotective properties (Rasul et al., 2013). Although a direct link between antioxidants and the modulation of cancer immunoediting has not been fully established, evidence suggests that flavonoids may be able to modulate immunoediting processes like restoring cancer immune surveillance which may be helpful in eradicating cancer cells (Bhattacharyya et al., 2010; Bose et al., 2015). Additionally, such flavonoids have been demonstrated to play a crucial role in mitigating the cardiotoxic effects inferred by Dox administration (Bast et al., 2007; Sun et al., 2007; Chang et al., 2019). In the case of Pin, its ability to regulate inflammatory cytokines, improve mitochondrial function whilst inhibiting platelet aggregation and mitigating apoptosis in the cardiac muscle makes it a suitable candidate in the prevention of Dox-induced cardiotoxicity (Saad et al., 2015; Shen, 2019). Pinocembrins’ cardioprotective potential is further endorsed by its vasorelaxant (Estrada et al., 2020) effects which could aid in the preservation of cardiac function by alleviating cardiac fibrosis and left ventricular dysfunction (Rasul et al., 2013; Borriello et al., 2019), which is a hallmark for Dox-induced cardiotoxicity. Literature suggests that H9c2 cells are a suitable model to study the effects of Dox-induced cardiotoxicity in an *in vitro* setting, as it has been shown that the major adverse effects reported in patients exposed to Doxcan be induced in these cells (Catanzaro et al., 2019; Dallons et al., 2020; Faridvand et al., 2020; Hu et al., 2020). Hence, the objective of the current study was to i) assess the prophylactic effect of Pin against the cardiotoxicity inferred by Dox administration using an H9c2 cardiomyoblast model, as well as ii) to evaluate the effect of Pin on the chemotherapeutic potential of Dox in an MCF-7 breast cancer cell line.

## Materials and Methods

### Preparation of Pinocembrin, Doxorubicin, and Dexrazoxane

Stock solutions of Pinocembrin (MW: 256.25 g/mol) (Pin, 10 mM, BioPharm™, New Zealand) and Dexrazoxane (MW: 268.269 g/mol) (Dex, 1 mM) (Sigma-Aldrich, St Louis, MO, USA) were prepared in Dimethyl sulfoxide (DMSO, Sigma-Aldrich, Saint Louis, MO, USA). DMSO concentration was kept below 0.0001%. Doxorubicin hydrochloride (MW: 579.98 g/mol) (Dox, 1 mM) was prepared in tissue culture (TC) grade water. Thereafter, final concentrations of 1 µM (Pin), 20 µM (Dex) and 2 µM (Dox) were prepared in Dulbecco’s modified Eagle medium without phenol red (DMEM, Lonza, Walkersville, MD, USA) supplemented with 2% fetal bovine serum (FBS, Thermo Fisher Scientific, Waltham, MA, USA) and then filter sterilized using 0.22 µM syringe filter systems prior to commencing treatment. Doses for Dox and Dex were derived from the clinically recommended therapeutic ratio of 1:10 (Rharass et al., 2016), while the concentration used for Pin was extrapolated from our preliminary dose response findings.

### H9c2 Cardiomyoblasts

Rat heart ventricular derived H9c2 cardiomyoblasts are immortalized cells with a cardiac phenotype and have been extensively used as a screening tool for novel therapeutic agents and to investigate the effects of Dox-induced cardiotoxicity (Dallons et al., 2020; Hu et al., 2020). The H9c2 cells were purchased from the American Type Culture Collection (ATCC, catalogue number CRL-1446). Briefly, H9c2 cells were cultured in Dulbecco’s modified Eagle medium (DMEM, Lonza, Walkersville, MD, USA) supplemented with 10% FBS under standard tissue culture (TC) conditions (37 °C, 95% humidified air and 5% CO_2_). Cells were passaged regularly at 80–90% sub-confluence and were seeded in 96-well (0.8 x 10^5^ cells/well), 24-well (1 x 10^5^ cells/well), or 6-well plates (2 x 10^5^ cells/well). To attain the most therapeutic dose, H9c2 cells were exposed to log concentrations (0.01–1,000 µM) of Pin, for 6 days. The therapeutic dose was determined as the dose that would increase cell viability in the H9c2 cells. Thereafter, chronic cardiotoxicity was induced by exposing the H9c2 cells with 2 µM Dox. The prophylatic effect of Pin against Dox-induced cardiotoxicity was determined by co-treating the cells with Dox (2 µM) plus Pin (1 µM). H9c2 cells co-treated with Dox (2 µM) plus Dex (20 µM) served as a positive control. Untreated cells served as the control group. H9c2 cells were treated every second day for 6 days and experiments conducted on day 7.

### MCF-7 Breast Cancer Cells

Human metastatic breast cancer derived MCF-7 cells are estrogen receptor positive and are often used *in vitro* to study estrogen receptor positive breast cancers (Vantangoli et al., 2015). MCF-7 cells were purchased from the American Type Culture (ATC, catalogue number HTB-22) and cultured in DMEM supplemented with 10% FBS, under standard TC conditions. Similar to the H9c2 cells, MCF-7 breast cancer cells were exposed to log concentrations (0.01–1,000 µM) of Pin to ensure that the selected therapeutic dose for the H9c2 cells would not stimulate cell proliferation in the MCF-7 cells. Thereafter, as a proof concept, cells were treated with Dox (2 µM). To assess whether the chemotherapeutic effect of Dox was maintained, cells were also co-treated with Dox (2 µM) plus Pin (1 µM) as well as Dox (2 µM) plus Dex (20 µM). To assess the anti-cancer effect of Pin, cells were treated with 1 µM Pin alone. Untreated cells served as the control. Cells were treated every second day for 6 days and experiments conducted on day 7.

### Assessment of Cell Viability

Cell viability was assessed by conducting an MTT [3-[4,5-dimethylthiazole-2-yl]-2,5-diphenyltetrazolium bromide dye (Sigma-Aldrich, St Louis, MO, USA)] assay using an in-house protocol. Briefly, after 6 days of treatment, the treatment media was aspirated and the H9c2 cells were stained with 100 µl of the MTT dye (2 mg/ml MTT dissolved in Dulbecco’s phosphate-buffered saline). To facilitate the reaction, stained cells were incubated for 30 minutes (min), under standard TC conditions. Thereafter, the MTT dye was discarded and replaced with 200 µl DMSO and 25 µl ice cold Sorenson’s buffer. Cells were then incubated for 5 min at room temperature (RT), before reading the absorbance (570 nm) with a BioTek^®^ ELx600 plate reader (Bio-Tek Instruments, Inc., Winooski, VT, USA using Gen 5^®^ software).

### Determination of Metabolic Activity

Cytoplasmic levels of ATP production was quantified using a ViaLight plus ATP assay kit (Lonza, Walkersville, MD, USA) as per the manufacturer’s instructions. Briefly, 140 µl of treatment media from H9c2 and MCF-7 cells, previously seeded in white 96 well plates, was discarded leaving a volume of 60 µl in each well. To this, an additional 60 µl of the ATP cell lysis reagent was pipetted into each well, followed by 10 min incubation under standard TC conditions. Subsequently, protein concentration was quantified as described in 2.4.1. Next, 100 µl of the ATP monitoring reagent was pipetted into cell lysates and then incubated for an additional 5 min RT. Luminescence, as measurement of metabolic activity, was measured on the BioTek^®^ FLx800 plate reader using the Gen 5 software^®^ (Bio-Tek Instruments, Inc., Winooski, VT, USA). Cellular metabolic activity was normalized against the protein data.

### Quantifying Protein Concentration

Total protein concentration was measured using the Bradford assay which relies on the formation of a complex between Coomassie brilliant blue G-250 dye and proteins in solution. Briefly, 5 µl of cell lysates from previously treated cells was transferred into a new 96 well plate. Thereafter, 180 µl of the Bradford reagent was pipetted into the cell lysates and then incubated at RT for 5 min. The protein concentration was determined by the amount of blue ionic dye measured by the absorbance of the cell lysate solution at 595 nm using the Gen 5 software^®^ (Bio-Tek Instruments, Inc., Winooski, VT, USA).

### Quantification of Reactive Oxygen Species (ROS) Production

Intracellular production of reactive oxygen species (ROS) was determined using an OxiSelect^TM^ Intracellular ROS assay kit (Cell Biolabs, San Diego, USA). After 6 days of treatment, media was aspirated and the H9c2 cells were washed with pre-warmed Dulbecco’s phosphate-buffered saline (DPBS, Lonza, Walkersville, MD, USA) before being stained with 100 µl of DCFH-DA (20 μM) staining solution. Thereafter, the stained cells were incubated under standard TC conditions for 30 min. Following incubation, the DCFH-DA staining solution was discarded and the cells were reconstituted in 100 µl of Hank’s Balanced Salt Solution (HBSS, Sigma-Aldrich, St Louis, MO, USA). ROS activity was then quantified by measuring the fluorescent intensity of DCFH-DA at excitation and emission wavelengths of 485± 20 nm/528±20 nm using a BioTek FLx800 plate reader and analysed with Gen 5^®^ software.

### The Assessment of Lipid Peroxidation

Lipid peroxidation was quantified by measuring the production of malondialdehyde (MDA) using an OxiSelect™ thiobarbituric acid (TBA) reactive substances (TBARS) assay Kit (Cell Biolabs, San Diego, USA). To account for the detached cells following treatment exposure, the supernatant of cells previously seeded in 6 well plates was collected into 15 ml tubes before washing the cells with pre-warmed DPBS. The cells were then trypsinized, centrifuged and collected into 2 ml eppendorf tubes at a cell suspension of approximately 1 x 10^7^. The cell suspension was then re-suspended in PBS containing 1X butylated hydroxytoluene (BHT) before being homogenized with an ice cold Qiagen TissueLyser block (Qiagen, Hilden, Germany) for 1 min. A dilution series (125 μM–0 μM) of MDA standards were prepared by diluting the MDA Standard, provided in the TBARS kit, in deionized water. Subsequently, 100 µl of the cell lysates and MDA standards were transferred into new eppendorf tubes followed by the addition of 100 µl of sodium dodecyl sulfate lysis solution and mixed thoroughly. The samples were then incubated for 5 mins at RT. Thereafter, 250 μl of TBA reagent was added to each sample and then incubated at 95°C on a heating block for 60 min. Following incubation, the tubes were chilled to RT in an ice bath for 5 min and the samples were centrifuged at 3,000 rpm for 15 min. Thereafter, 150 µl of the MDA standard and sample supernatant was transferred into a 96 well black fluorescence plate. Fluorometric measurement was read at an excitation of 540 nm and emission of 590 nm.

### Quantification of Superoxide Dismutase Activity

The activity of the antioxidant superoxide dismutase (SOD) was measured using a colorimetric SOD activity assay kit (Abcam, Pretoria, SA) as per the manufacturer’s instructions. Briefly, cell suspensions were prepared as described in section 1.6. The cells were lysed with 100 µl of 0.1 M trizma/hydrochloride (Tris/HCl), [comprising of 0.5% Triton X-100, 5 mM β-methylphenethylamine (β-ME) and 0.1 mg/ml phenylmethylsulfonyl fluoride (PMSF) at a pH of 7.4] and then transferred into 2 ml eppendorf tubes. Cell lysates were then centrifuged at 15,000 x g for 5 min at 4°C. Thereafter, 20 µl of the supernatants was pipetted into a new 96 well assay plate and to this, 200 µl of the 2-(4-iodophenyl)-3-(4-nitrophenyl)-5-(2,4-disulfo-phenyl)-2H-tetrazolium, monosodium salt (WST) working solution and 20 µl of the enzyme working solution was added to the supernatants. The plates were then incubated at 37 °C for 20 min. After incubation, SOD activity was measured on the Biotek^®^ ELX 800 plate reader (Gen 5® software) at an absorbance rate of 450 nm.

### Quantification of Total Glutathione Content

The GSH/GSSH-Glo™ assay kit (Promega, Madison, Wilsconsin, USA) is a quantitative assay that encompasses both reduced (GSH) and oxidized glutathione (GSSG) and was used to quantify total glutathione content of H9c2 cells and MCF-7 breast cancer cells seeded in white 96 well plates, as per the manufacturer’s protocol. Following treatment, media was aspirated and then cells were lysed with 50 µl of either GSH or GSSG lysis reagent. To facilitate the lysis reaction, plates were incubated at RTon an orbital shaker for 5 min. Thereafter, 50 µl of the Luciferin generation reagent was added to each well containing the cell lysates and then incubated under standard TC conditions for 30 min. After incubation, 100 µl of the luciferin detection reagent was pipetted into each well and then equilibrated for 15 min under standard TC conditions. Relative luminescence unit (RLU) was measured using the SpectraMax i3x^®^Multi-Mode Microplate Reader. Total GSH content was measured as the ratio of GSH/GSSG using the following formula:

$$Ratio\;of\;GSH/GSSG=\frac{\left(Net\;treated\;GSH\;RLU-Net\;treated\;GSSG\;RLU\right)}{\left(Net\;treated\;GSSG/2\right)}$$

### Mitochondrial Bioenergetics

The oxygen consumption rate (OCR) and extracellular acidification rate (ECAR) of intact H9c2 cells were measured using a Seahorse XF96 extracellular flux analyzer (Seahorse Bioscience, Billerica, MA, USA). Briefly, 80 μL of single-cell suspensions of H9c2 cells were plated in XF96 cell culture microplates (Seahorse Bioscience) at a cellular density of 1 x 10^4^ and 5 x 10^3^ per well, respectively. After 48 h, cells were treated as per the treatments conditions described in section 1.2 for 6 days. On the day of the assay, cells were incubated in base assay medium supplemented with 2 mM glutamine, 10 mM glucose, and 1 mM pyruvate for 1 h, prior to the OCR and ECAR measurements using the XF Cell Mito Stress Kit (Seahorse Bioscience), as per the manufacturer’s instructions. Mitochondrial OCR was measured over a period of 86 min over which time 1 μM oligomycin (ATP-synthase inhibitor), 0.75 μM FCCP (a mitochondrial uncoupler), and 5 μM rotenone (complex I inhibitor) plus 5μM antimycin A (complex III inhibitor) were sequentially added to each well at specified time points. All compound injection solutions were prepared at a concentration 10x higher than the required finale concentration.

Data were normalized according to total protein content to control for the variation in cell number between Dox and Pin treated cells as well as control groups. On completion of the XF assay, H9c2 cells were lysed with 100 μl of cell lysis reagent and protein concentration determined using the Bradford reagent. Protein concentrations were derived by reference to a bovine serum albumin (BSA) standard curve. The OCR data were expressed as pmol/min/mg protein and ECAR as mpH/min/mg protein.

### Evaluation of Mitochondrial Membrane Potential

Mitochondrial membrane potential (MMP) was assessed by staining cells with the cationic dye 5,5’,6,6’-tetrachloro-1,1’,3,3-tetraethylbenzimidazolyl-carbocyanine iodide (JC-1, Sigma-Aldrich, St Louis, MO, USA) as per the manufactures instructions. Briefly, treatment media from H9c2 and MCF-7 cells, previously seeded in black 96 well plates, was aspirated and the cells were washed twice with pre-warmed DPBS. Thereafter, cells were stained with 100 µl of JC-1 dye at a concentration of 15.4 µM followed by a 45 min incubation under standard TC conditions. After incubation, the JC-1 dye was discarded and the cells washed with 100 µl DPBS. Mitochondrial integrity was quantified by measuring the fluorescence intensity of J-aggregates, which is an indication of high MMP and is emitted as red fluorescence at ~590 nm, and JC-1 monomers, which indicates low MMP and is emitted as green fluorescence at ~529 nm, using a BioTek® FLx 800 plate reader. Data was normalized by fluorescence imaging using a Nikon inverted fluorescence microscope.

### Evaluation of Apoptosis: Quantification of Annexin V and Propidium Iodide

Annexin V-FITC (Invitrogen, Carlsbad, CA, USA) and propidium iodide (PI, Sigma-Aldrich, St Louis, MO, USA) were used to detect early and late apoptosis as well as necrosis in H9c2 and MCF-7 cells, respectively. Briefly, cells that were previously treated in 24 well plates were harvested by collecting the supernatant (containing the detached cells) into 15 ml centrifuge tubes and by trypsinization, i.e. 200 µl trypsin was pipetted to the cells and then incubated for 5 min under standard TC conditions. Trypsinization was deactivated by adding 500 µl of DPBS supplemented with 10% FBS (staining buffer). The combined cell suspension (supernatant plus the trypsinized cells) was centrifuged at 300 g for 5 min. Subsequently, the supernatant was decanted and the cells were re-suspended in 150 ul of staining buffer before being transferred into 2 ml eppendorf tubes. The H9c2 and MCF-7 cells were then stained with 1.5 μl of Annexin V and 1 μl of PI (2 µg/ml) staining solution. Stained H9c2 cells were incubated in the dark for 10 min, while the MCF-7 cells were incubated in the dark for 20 min. After incubation, cells were acquired using a BD Accuri C6 flow cytometer (BD Biosciences) using the BD Accuri C6 Annexin V-FITC/PI template. Live, early and late apoptotic, and necrotic cells were analyzed with the BD Accuri C6 software using the FITC signal detector FL1 (excitation= 488 nm; emission = 530 nm) for Annexin V positive cells and FL3 detector (excitation= 488 nm; emission = 670/LP) for PI positive cells.

### Statistical Analysis

All data was statistical analyzed using GraphPad Prism software version 5.0 (GraphPad Software, Inc. La Jolla, USA). The data are represented as the mean ± standard error of mean (SEM) of three independent biological experiments, with each experiment containing at least six replicates. Comparisons between treatment groups were performed using one-way analysis of variance (ANOVA), followed by a Tukey post hoc test and a student’s t-test. A p value of ≤0.05 was considered as statistically significant.

## Results

### Cytotoxicity Effect of Pinocembrin

**Figure 1:**
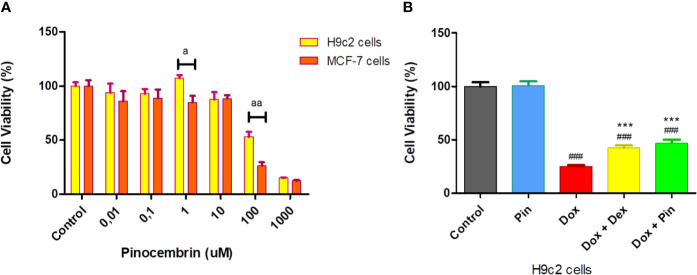
The effect of pinocembrin (Pin) and the co-administrative effect of doxorubicin (Dox) plus Pin on cell viability. **(A)** Cytotoxicity effect of Pin on H9c2 cardiomyoblasts and MCF-7 breast cancer cells. **(B)** Co-administrative effect of Dox plus Pin on Dox-induced cardiotoxicity. Briefly, H9c2 and MCF-7 cells were exposed to log concentration (0.01–1,000 µM), of Pin for 6 days to attain the most therapeutic dose. Thereafter, chronic cardiotoxicity was induced by subjecting the H9c2 cells to Dox (2 µM) for 6 days. Cells were also co-treated with Dox (2 µM) plus Pin (1 µM) and Dox (2uM) plus Dexrazoxane (Dex) (20 µM) or treated with Pin (1 µM) for the same treatment duration. Untreated cells served as the control. The results were analyzed using One-way Anova, student t-tests, or non-parametric tests where applicable. Data are presented as the mean ± SEM of 5 biological experiments with 3 technical repeats (n=15). Significance is indicated as ^a^p ≤ 0.05, ^aa^p ≤ 0.01, versus the H9c2 cells, ^###^p ≤ 0.001 versus the control and ^***^p ≤ 0.001 versus Dox.

To attain the most effective therapeutic dose of Pin, cell viability, using the MTT assay, was quantified to ensure that the selected dose (i) would not be toxic to the H9c2 cardiomyoblasts and (ii) would not increase the proliferation of the MCF-7 breast cancer cells. Our findings showed no significant change in the viability of the H9c2 and MCF-7 cells from concentrations 0.01–10 µM after 6 days of treatment versus the control group. However, a significant increase in cytotoxicity was observed at 100 and 1,000 µM in both cell lines. When we compared the effect of Pin between the two cell lines, we observed a significant difference in viability at a dose of 1 µM, as demonstrated by an increase in the number of viable H9c2 cells with a concomitant decrease in viable MCF-7 cells. Based on these findings a dose 1 µM was selected for all subsequent experiments (**Figure 1A**).

Next, we assessed the co-administrative effect of Dox plus Pin on the H9c2 cells against Dox-induced cytotoxicity. A significant loss in cell viability was observed in Dox-treated cells versus the control group (24.89 ± 1.38% versus 100 ± 3.92%, p = 0.0001). No noticeable effect was observed in normal cells treated with Pin (100.80 ± 3.93% compared to 100.00 ± 3.92%, p ≥ 0.05). However, co-treatment with Dox plus Pin significantly attenuated (46.93 ± 3.32%, p ≤ 0.001) Dox-induced cytotoxicity. Cytoprotection was also sustained in H9c2 cells co-treated with Dox plus Dex (42.27 ± 2.48%, p ≤ 0.001) when compared to cells treated with Dox alone (**Figure 1B**).

### Co-Treatment With Pinocembrin Attenuates Doxorubicin-Induced Oxidative Stress

**Figure 2:**
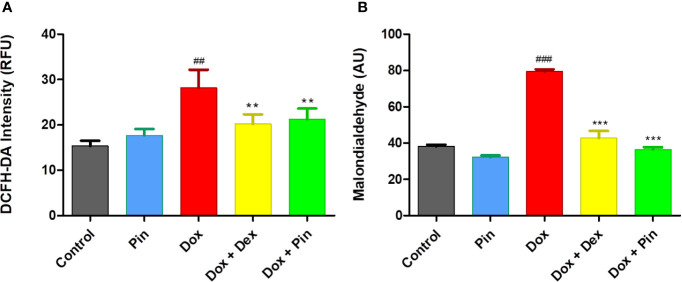
The effect of pinocembrin (Pin) on doxorubicin (Dox)-induced oxidative stress. **(A)** ROS production was quantified using a fluorogenic dye (2,7-dichlorofluorescin diacetate (DCFH-DA) which measures hydroxyl and peroxyl radicals. **(B)** Lipid peroxidation was quantified by measuring the malondialdehyde (MDA) which reacts with thiobarbituric acid (TBA) to generate an MDA-TBA adduct. H9c2 cells were treated with Dox (2 μM), Pin (1 μM), Dox (2 μM) plus Pin (1 μM) and Dox plus Dex (20 μM) for 6 days. The results were analyzed using One-way Anova, student t-tests, or non-parametric tests where applicable. Data are shown as the mean ± SEM of 3 biological experiments with 3 technical repeats (n=9). Significance is indicated as ^##^p ≤ 0.01, ^###^p ≤ 0.001 versus the control;^**^p ≤ 0.01, ***p ≤ 0.001 versus Dox. Relative fluorescence units (RFU). Arbitrary units (AU).

In cardiac cells, Dox triggers mitochondria-dependent apoptosis primarily by inducing oxidative stress. Accordingly, Dox-induced ROS production in the H9c2 cells was quantified with DCFH-DA fluorogenic dye. As demonstrated in **Figure 2A**, the level of ROS activity increased significantly in Dox-treated H9c2 cells when compared to the control group (36.96 ± 4.35 compared to 18.43 ± 1.28; p ≤ 0.01). Whereas, co-treatment with Dox plus Pin (28.78 ± 3.03, p ≤0.01) was able to significantly attenuate Dox-induced oxidative stress. As an oxidative damage parameter, lipid peroxidation, as demonstrated by malondialdehyde (MDA) was found to be significantly augmented in the Dox group (79.50 ± 1.21, p ≤ 0.001) but, was expressively alleviated in cells co-treated with Dox plus Pin (36.50 ± 1.33; p ≤ 0.001) (**Figure 2B**). Additionally, cells co-treated with Dox plus Dex also presented with reduced ROS production (25.09± 2.45; p ≤ 0.01) and MDA content (42.83 ± 3.85; p ≤ 0.001). No notable effect in ROS activity (20.69± 1.54, p = 0.28) and MDA content (32.17 ± 1.07; p = 0.06) was observed in cells treated with Pin alone.

### Pinocembrin Ameliorates Antioxidant Enzyme Activity in H9c2 Cells

**Figure 3:**
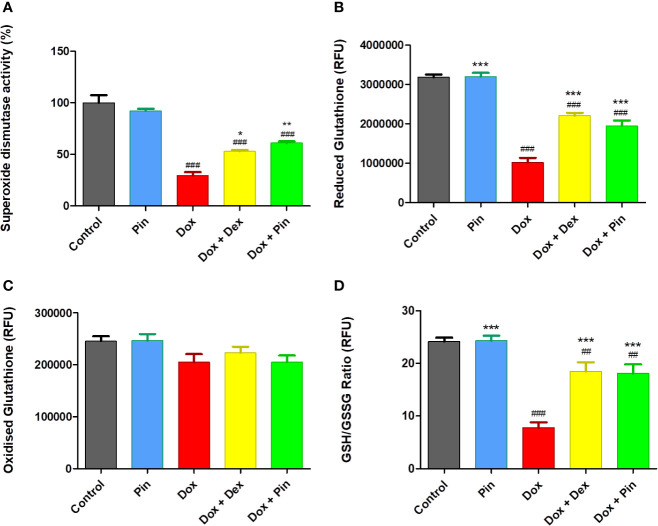
The co-administrative effect of doxorubicin (Dox) plus pinocembrin (Pin) on the antioxidant capacity of H9c2 cells. **(A)** Superoxide dismutase activity, **(B)** reduced glutathione (GSH), **(C)** oxidized glutathione (GSSG) and **(D)** total GSH content (ratio of GSH/GSSG). H9c2 cells were treated with Dox (2 μM), Pin (1 μM), Dox (2 μM) plus Pin (1 μM) and Dox plus Dex (20 μM) for 6 days The results were analyzed by One-way Anova, student t-tests, or non-parametric tests where applicable. Data are shown as the mean ± SEM of 3 biological experiments with 3 technical repeats (n=9). Significance is indicated as ^##^p ≤ 0.01, ^###^p ≤ 0.001 versus the control; ^*^p ≤ 0.05; ^**^p ≤ 0.01, ^***^p ≤ 0.001 versus Dox.

As a confirmation of Dox-induced oxidative stress, a significant decrease in superoxide dismutase (SOD) activity (29.61 ± 3.14 versus 100.00 ± 7.41% to, p ≤ 0.001), reduced glutathione (GSH) and total glutathione content (ratio of GSH/GSSG) (7.13 ± 1.01 versus 24.21 ± 0.712; p = 0.001) was demonstrated by Dox-treated H9c2 versus the control (**Figures 3A–D**). However, Dox-induced oxidative stress was significantly ameliorated by Dox plus Pin as presented by increased SOD activity (61.11 ± 1.57%, p ≤ 0.01) and total glutathione content (17.43 ± 1.77, p ≤ 0.001). Additionally, cells co-treated with Dox plus Dex also had increased SOD activity (52.95 ± 1.35; p ≤ 0.05) and GSH/GSSG content (18.37 ± 1.57, p ≤ 0.001). Treatment with Pin had no notable effect on the antioxidant levels [92.22 ± 2.02 (SOD) and 24.33 ± 0.08 (GSH/GSSG)] (**Figures 3A, D**).

### Effect of Pinocembrin on the Antioxidant Capacity of MCF-7 Cells

**Figure 4:**
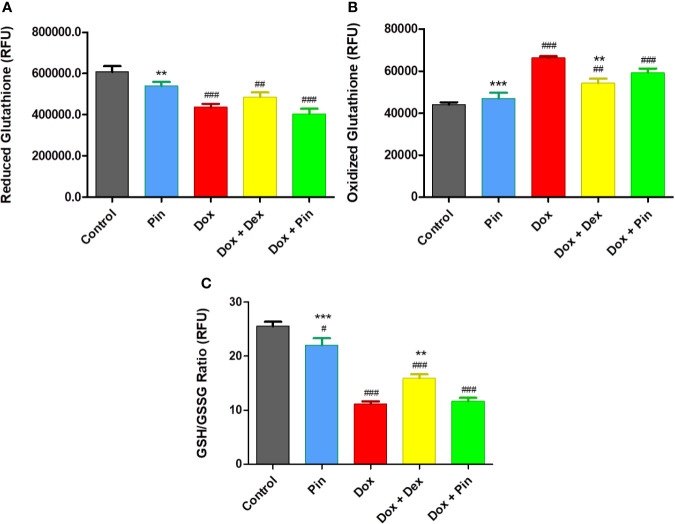
**(A)** Reduced glutathione (GSH), **(B)** Oxidized gutathione (GSSG), **(C)** Total GSH content (ratio of GSH/GSSG). The co-administrative effect of doxorubicin (Dox) plus pinocembrin (Pin) on total glutathione content. To induce cytotoxicity, MCF-7 breast cancer cells were treated with Dox (2 µM), Pin (1 µM) or co-treated with Dox (2 µM) plus Pin (1 µM) and Dox (2 µM) plus Dex (20 µM) for 6 days. Untreated cells served as the control. The results were analyzed using One-way Anova, student t-tests, or non-parametric tests where applicable. Data are presented as the mean ± SEM of 3 biological experiments with 3 technical repeats (n=9). Significance is indicated as ^#^p ≤ 0.05, ^##^p ≤ 0.01, ^###^p ≤ 0.001 versus the control; ^**^p ≤ 0.01, ^***^p ≤ 0.001 versus Dox.

In cancer studies, high levels of antioxidants have been shown to offer a survival advantage in *in vitro* and *in vivo* models. Therefore, the notion that ROS are bad and antioxidants are good depends, to some extent, on context. Here, Dox-treated MCF-7 breast cancer cells were primarily characterized by a substantial increase in GSSG levels and a considerable reduction in GSH and GSH/GSSG content (11.17 ± 0.48 versus 25.55 ± 0.81; p ≤ 0.001) (**Figures 4A–C**). While Dox plus Pin improved the antioxidant capacity of the H9c2 cells (**Figures 3A–D**), the co-treatment had no significant effect on the MCF-7 cells total glutathione content (11.66 ± 0.633; p ≤ 0.55) versus the cells treated with Dox alone (**Figures 4A, C**). These findings suggest that Dox plus Pin may facilitate the accumulation of ROS in the metastatic MCF-7 cells. In addition, cells treated with Pin presented with a significant reduction in total glutathione content (22.03 ± 1.27; p ≤ 0.001) when compared to the control. Interestingly, co-treatment with Dox plus Dex had a significant effect on Dox-induced oxidative stress as demonstrated by the increased antioxidant status (15.96 ± 0.68; p ≤ 0.01) of the metastatic MCF-7 breast cancer cells.

### Pinocembrin Improves Mitochondrial Bioenergetics in H9c2 Cells

**Figure 5:**
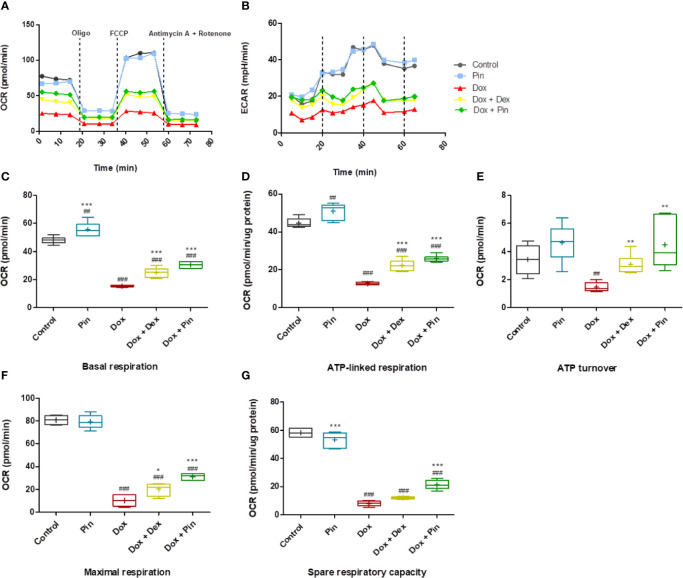
Co-treatment with doxorubicin (Dox) plus pinocembrin (Pin) improves mitochondrial respiration. **(A)** Mitochondrial oxygen consumption rate (OCR) **(B)** extracellular acidification rate (ECAR), **(C)** basal respiration, **(D)** ATP-linked respiration, **(E)** ATP turnover, **(F)** maximal respiration and **(G)** spare respiratory capacity Basal respiration was acquired after subtraction of non-mitochondrial respiration. ATP turnover measured by ATP-linked respiration subtracted from the basal OCR. Maximum respiration calculated by non-mitochondrial respiration subtracted from FCCP-stimulated OCR. Coupling efficiency was calculated as the fraction of basal mitochondrial OCR used for ATP synthesis (ATP-linked OCR/basal OCR). Results were analyzed using One-way Anova, student t-tests, or non-parametric tests where applicable. Data are presented as the mean ± SEM of 3 biological experiments with 6 technical repeats (n=9). Significance is indicated as ^##^p ≤ 0.01, ^###^p ≤ 0.001 versus the control; *p < 0.05, ^**^p ≤ 0.01, ^***^p ≤ 0.001 versus Dox.

OCR and extracellular acidification rate (ECAR) were evaluated to ascertain whether the mitochondria of Dox-treated H9c2 cells had defective mitochondrial bioenergetics. Real-time OCR revealed that basal respiration (15.37 ± 0.25 relative to 48.06 ± 1.02; p ≤ 0.05), demonstrating the quantity of all physiological mitochondrial OC, was significantly reduced in the mitochondria’s of Dox-treated cells which represents lower respiratory function relative to the control (**Figures 5A, C**). Similarly, this reduction was reflected in the ECAR (11.99 ±0.82 versus 33.34 ± 3.14) of the Dox-treated cells (**Figure 5B**). However, co-treatment with Dox plus Pin was able to attenuate this effect (30.46 ± 0.91 (OCR) and 20.67 ± 0.92 (ECAR); p ≤ 0.001) (**Figures 5A–C**). While Dox significantly decreased ATP turnover (1.46 ± 0.15; p ≤ 0.01) (**Figure 5E**), which is measured by ATP-linked respiration (**Figure 5D**) subtracted form basal OCR (**Figure 5C**), co-treatment with Dox plus Pin was able to improve mitochondrial ATP production (4.49 ± 0.61; p ≤ 0.01). In addition, the maximal respiration (MR, 30.96 ± 1.29; p ≤ 0.001), as calculated by non-mitochondrial respiration subtracted from FCCP-stimulated OCR, and spare respiratory capacity (SRC, 21.22 ± 1.35; p ≤ 0.001) was significantly ameliorated in cells co-treated with Dox plus Pin in comparison to the Dox-treated cells (10.09 ± 1.95 (MR) and 7.95 ± 0.76 (SRC) (**Figures 5F, G**). The state _apparent_, which is an estimation of the relative mitochondrial work used by cells under basal conditions, was significantly low in Dox-treated cells suggesting a reduction in the overall flux of electrons through the respiratory chain (**Table 1**). However, co-treatment with Dox plus Pin was able to enhance the state _apparent_ of these cells. We then assessed the respiratory control ratio (RCR), which represents the tightness of the coupling between respiration and oxidative phosphorylation, where the RCR value is sensitive to alterations in substrate oxidation and proton leak, but not ATP turnover. Here, co-treatment with Dox plus Pin alleviated Dox-induced impaired RCR value in the H9c2 cells indicating an elevated potential for substrate oxidation and ATP turnover (**Table 1**). We further observed a substantial increase in coupling efficiency after co-treatment with Dox plus Pin, which demonstrates a much higher proportion of oxygen consumed to stimulate ATP production compared with that driving proton leak (**Table 1**). Interestingly, co-treatment with Dox plus Pin was more effective at improving mitochondrial oxidative phosphorylation than co-treatment with Dox plus Dex (**Figures 5D–G**).

**Table 1:** Normalized respiratory flux control ratios.

Normalized respiratory flux ratios	H9c2 cells treatment				
	Control	Pin	Dox	Dox + Dex	Dox + Pin
**State _apparent_**	3.60±0.01	3.49±0.02	3.23±0.05^###^	3.20±0.09^##^	3.38±0.02^*^
**Respiratory control ratio (RCR)**	39.27±3.94	33.67±1.84^**^	15.85±2.64^##^	15.44±1.98^###^	23.85±3.27^##^
**Coupling efficiency**	0.93±0.01	0.91±0.02^***^	0.82±0.01^###^	0.87±0.01^##^	0.88±0.01^*^

The state _apparent_, respiratory control ratio (RCR) and coupling efficiency of H9c2 cells in the presence or absence of Dox, Pin, Dox plus Pin and Dox plus Dex were derived from the mitochondrial parameters presented in Figure 5. Data represents mean ± SEM; n=6. Significance is indicated as ^##^p ≤ 0.01, ^###^p ≤ 0.001 versus the control; ^*^p ≤ 0.05, ^**^p ≤ 0.01, ^***^p ≤ 0.001 versus Dox.

### Effect of Pinocembrin on Doxorubicin-Induced Mitochondrial Depolarization

**Figure 6:**
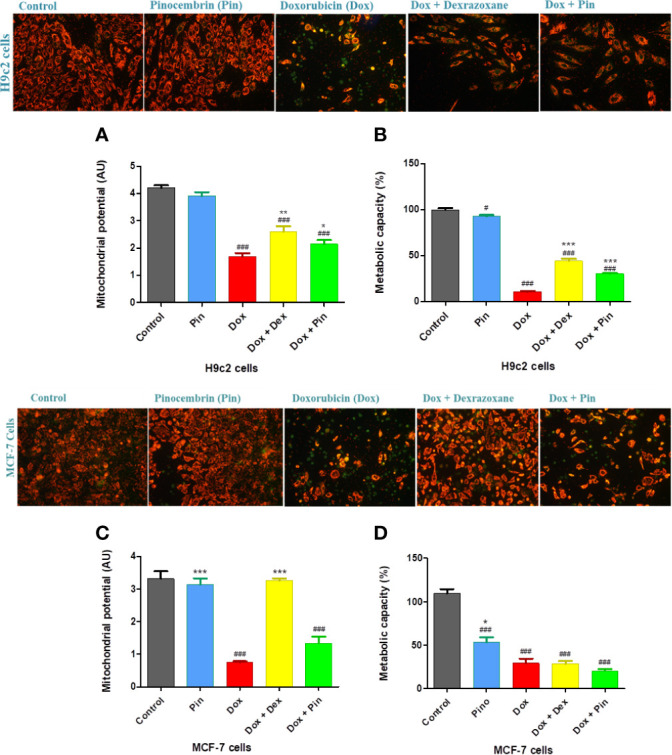
The co-administrative effect of doxorubicin (Dox) plus pinocembrin (Pin) on the mitochondrial integrity of the H9c2 cardiomyoblasts and MCF-7 breast cancer cells. **(A)** JC-1 staining and mitochondrial membrane potential (MMP) of H9c2 cells, **(B)** metabolic activity of H9c2 cells, **(C)** JC-1 staining and MMP of MCF-7 cells and **(D)** metabolic capacity of MCF-7. Results were analyzed using One-way Anova, student t-tests, or non-parametric tests where applicable. Data are shown as the mean ± SEM of 3 biological experiments with 4 technical repeats (n=12). Significance is indicated as^ #^p ≤ 0.05, ^###^p ≤ 0.001 versus the control; ^*^p ≤ 0.05, ^**^p ≤ 0.01, ^***^p ≤ 0.001 versus Dox.

Considering that mitochondrial membrane dysfunction triggered by Dox plays a pivotal role in the development of Dox-induced cardiomyopathy, we assessed whether co-treatment with Dox plus Pin would preserve mitochondrial function. JC-1 staining of Dox-treated H9c2 cells revealed mitochondrial deformities with loss in structural integrity as demonstrated by increased J monomers, representing loss in mitochondrial membrane potential (MMP) (1.69 ± 0.13 versus 4.21 ± 0.11; p ≤ 0.001), versus the control group (**Figures 6A, C**). Loss in MMP (0.75 ±0.03 versus 3.3 ± 0.22; p ≤ 0.001) was also sustained in MCF-7 cells treated with Dox (**Figure 6C**). Consistent with these findings, the metabolic capacity of the H9c2 cardiomyoblasts (10.60 ± 0.83%; p ≤ 0.001) and MCF-7 breast cancer cells (29.12 ± 5.22; p ≤ 0.001) was significantly impaired following Dox exposure (**Figures 6B, D**). Conversely, while the MMP (2.14 ± 0.15, p ≤ 0.05)and metabolic status (26.82 ± 1.99%; p ≤ 0.001) of the H9c2 cells was preserved following Dox plus Pin exposure, MCF-7 cells presented with significantly depolarized mitochondria (1.33 ± 0.21; p ≤ 0.20) and decreased metabolic status (20.31 ± 2.11; p ≤ 0.32) (**Figures 6A–D**). Interestingly, co-treatment with Dox plus Dex not only improved the MMP (2.59 ± 0.21; p ≤ 0.01) and metabolic capacity (44.24 ± 2.08%, p ≤ 0.001) of the H9c2 cells but, it also prevented Dox-induced mitochondrial depolarization (3.25 ± 0.07; p ≤ 0.001) (**Figures 6A–D**).

### Co-Treatment with Pinocembrin Mitigated Doxorubicin-Induced Apoptosis

**Figure 7:**
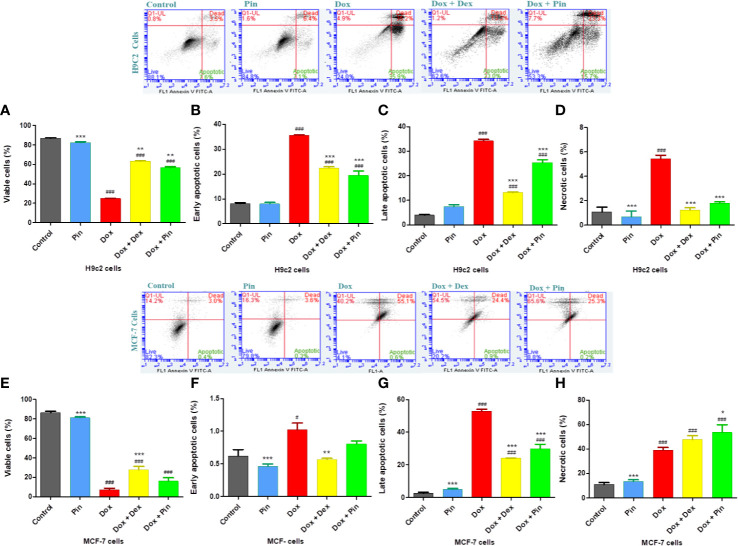
The effect of pinocembrin (Pin) on Doxorubicin-induced apoptosis. **(A)** Viable, **(B)** early apoptotic, **(C)** late apoptotic, and **(D)** necrotic H9c2 cells. **(E)** Viable MCF-7 breast cancer cells, **(F)** early apoptotic, **(G)** late apoptotic, and **(H)** necrotic MCF-7 cells. Annexin V and propidium iodide (PI) staining was evaluated using a BD Accuri C6. Upper right quadrant: Demonstrates cells showing Annexin V and PI positive and late apoptotic. Lower right quadrant: Cells that are Annexin V positive but are PI negative and are early apoptotic. Lower left quadrant: Consists of viable cells that are both Annexin V and PI negative. Upper left quadrant: Cells stained PI positive and are considered necrotic. The results were analyzed using One-way Anova, student t-tests, or non-parametric tests where applicable. Data are shown as the mean ± SEM of 3 biological experiments with 3 technical repeats (n=9). Significance is indicated as ^###^ p ≤ 0.001 versus the control;^**^p ≤ 0.01, ^***^p ≤ 0.001 versus Dox.

While cardiomyocyte apoptosis is a central mechanism underlying cardiac injury induced by Dox, cancer cell death is a key aspect of cancer therapy. As expected, the rate of early (35.53 ± 0.33%, p ≤ 0.001) and late (34.19 ± 0.61%; p ≤ 0.001) apoptosis as well as necrosis was significantly elevated in Dox-treated H9c2 cells and also presented with reduced number of viable cells (24.84 ± 0.46; p ≤ 0.001) (**Figures 7A, B, D, F, H**). Likewise, a decrease in cell viability (7.22 ± 1.55; p ≤ 0.001) with increased early (0.81 ±0.17; p ≤ 0.05) and late (52.93 ± 1.128; p ≤ 0.001) apoptosis, as well as necrosis (39.02 ± 2.22; p ≤ 0.001) was observed in MCF-7 exposed to Dox (**Figures 7A, C, E, G**). Given that Dox plus Pin attenuated Dox-induced early (15.740 ± 0.00%; p ≤ 0.001) and late (23.280 ± 0.00%; p ≤ 0.001) apoptosis, as demonstrated by an increase in the live population (50.540 ± 2.77%; p ≤ 0.001) of the H9c2 cells, here apoptosis (0.80 ± 0.05; p ≥ 0.05) triggered by Dox was sustained in MCF-7 cells co-treated with Dox plus Pin (**Figures 7A–H**). Interestingly, cancer cell death driven by Dox plus Pin was largely attributed to necrosis (53.54 ± 6.336; p ≤ 0.05) (**Figure 7I**). Conversely, although Dox plus Dex significantly decreased cardiomyocyte early (22.44 ± 0.50%; p ≤ 0.001) and late (13.16 ± 0.28%; p ≤ 0.001) apoptosis in H9c2 cells, our findings indicate that Dex significantly reduced the rate of apoptosis (24.01 ± 0.24; p ≤ 0.001) in the MCF-7 cells. This was further supported by the observed increase in the number of viable of cells (27.67 ± 3.49; p ≤ 0.001) (**Figure 7C**).

## Discussion

In the present study, we set out to investigate whether Pin could attenuate Dox-induced cardiotoxicity using an in H9c2 cardiomyoblast *in vitro* model. This model was selected based on current literature which indicates that H9c2 cells are a suitable *in vitro* model to study various forms of cardiotoxicity such as that inferred by Dox administration (Catanzaro et al., 2019; Dallons et al., 2020; Faridvand et al., 2020; Hu et al., 2020). Mechanistically, the pathophysiology of Dox-induced cardiotoxicity is said to be driven by increased oxidative stress, loss in mitochondrial membrane potential and aggravated apoptosis (Conklin, 2005; Carvalho et al., 2009; McGowan et al., 2017; Shabalala et al., 2017). Although there is an obvious need to investigate new cardioprotective agents, it is imperative to clarify whether or not newly developed therapeutic adjuncts interact with cancer therapy. Indeed, Yasueda and colleagues (2016) argued that while such therapeutic agents may mitigate the adverse effects of chemotherapy, they could antagonize the antitumor effects of drugs like Dox. Thus, we also assessed the effect of Pin on the efficacy of Dox in an MCF-7 breast cancer environment.

Generally, the cardiac muscle is considered a chief target for Dox-induced oxidative stress due to its naturally low antioxidant enzyme activity (Zhang and Wang, 2007). In the current study, chronic Dox exposure led to accelerated ROS production and lipid-peroxidation, which was concomitant with the observed reduction in SOD and GSH activity. We postulate that the perceived GSH deficiency could be due to GSH consumption in the interactions of Dox-induced free radicals with bio-membrane and the subsequent lipid peroxidation in the H9c2 cells (Zhang et al., 2019). However, Pin as a co-treatment was able to mitigate Dox-induced oxidative stress by boosting the antioxidant capacity of the H9c2 cells whilst decreasing ROS and MDA levels. As previously stated, cancer cells have the ability to take advantage of the upregulated antioxidant defense system to circumvent ROS-mediated tumor cell damage (Thyagarajan and Sahu, 2018). Here, co-treatment with Pin did not enhance the antioxidant activity of the Dox-treated MCF-7 cells, suggesting that Pin could be used as a potent ROS scavenger against Dox-mediated oxidative stress in the cardiac muscle without protecting the cancer cells. In contrast, MCF-7 cells co-treated with Dex presented with significantly improved antioxidant levels.

The high-energy-demand nature of the cardiac muscle requires that there be sufficient ATP production to sustain normal cardiac contraction (Werner et al., 2016). Studies have shown that Dox administration is associated with reduced metabolic activity and impaired cardiomyocyte viability (Chakrabarti et al., 2012). Similarly, data demonstrated in this study supported this notion as H9c2 cells subjected to chronic Dox conditions presented with reduced metabolic capacity leading to a significant loss in cell viability. Interestingly, Dox-induced loss in metabolic levels in MCF-7 cells was not affected by Pin administration. Pertaining to mitochondrial bioenergetics, in response to Dox exposure, we found that Pin was able to ameliorate mitochondrial OCR through enhanced oxidative phosphorylation in the H9c2 cells. These findings were in accordance with an *in vivo* study conducted by Wu and colleagues (2016) demonstrating that Dox significantly impaired mitochondrial function and ATP production in male rats. In addition, co-treatment with Pin significantly attenuated Dox-induced mitochondrial depolarization as indicated by the improved MMP of the H9c2 cells. Interestingly, while Pin preserved the loss in mitochondrial function and integrity in the MCF-7 cells, Dex significantly hindered the mitochondrial damage ensued by Dox on these cells.

In a clinical setting, Zhao and Zhang (2017) demonstrated that Dox administration activated cell death pathways in the hearts of cancer patients within hours of intravenous administration. Likewise, Subbarao and colleagues (2018) reported that H9c2 cardiomyoblasts treated with Dox had a significant increase in late apoptotic cells. Accordingly, our findings revealed that chronic Dox exposure significantly accelerates the rate of apoptosis in the cardiac and breast cancer cells. However, the results also showed that while Pin mitigates Dox-induced apoptosis in the H9c2 cells, it is able improve the apoptotic and necrotic status of inferred by Dox on the MCF-7 cells, which is a key aspect in cancer cell therapy. Although Dex is vastly used in chemotherapeutic regimens with Dox, data presented in this study suggests that Dex might indeed have some effect on Dox, as demonstrated by the decreased rate of metastatic MCF-7 apoptosis.

**Figure 8:**
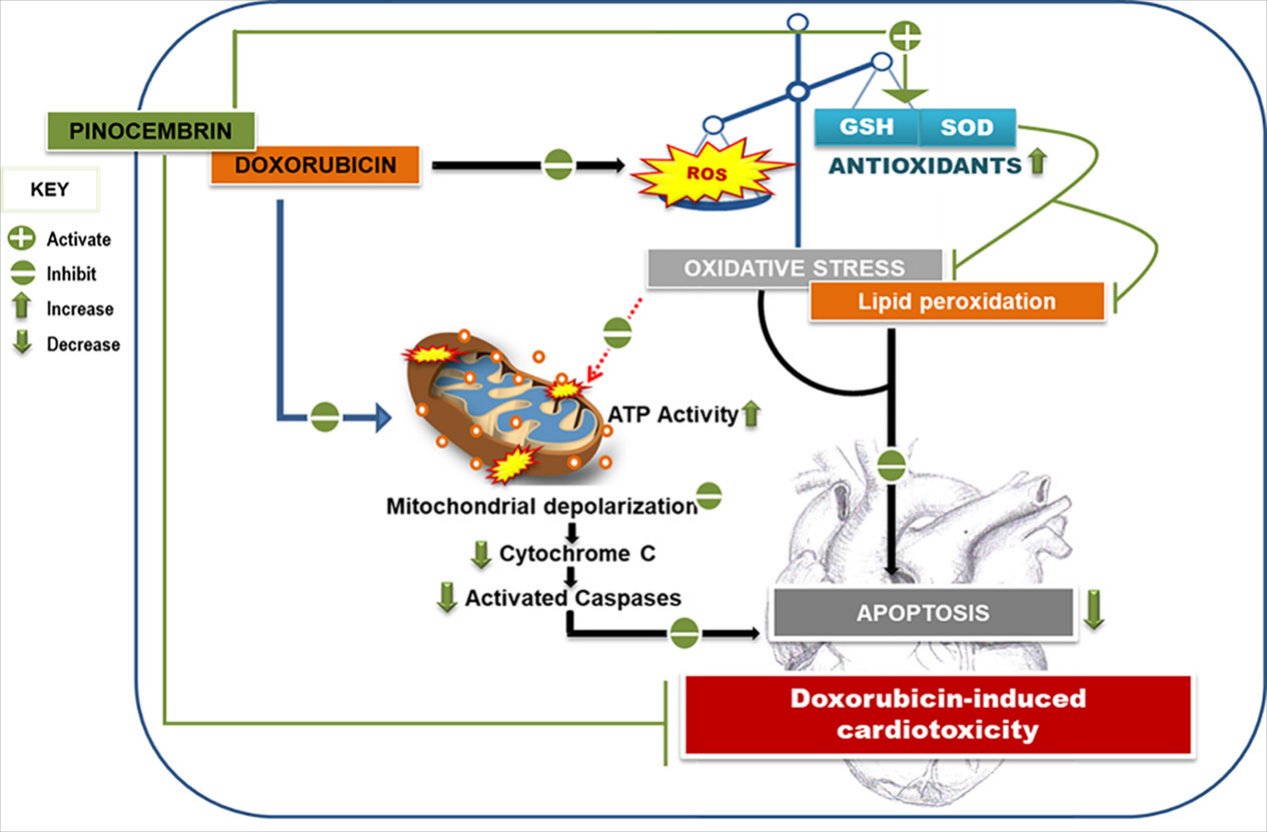
Pinocembrin attenuates Doxorubicin-induced cardiotoxicity. Pinocembrin as adjunct to the chemotherapeutic drug Doxorubicin protects H9c2 cardiomyoblasts from Doxorubicin-mediated oxidative stress and lipid peroxidation by boosting the activity of superoxide dismutase and the ratio of reduced glutathione to oxidized glutathione content. This increase in antioxidant capacity thereby ameliorates the mitochondrial membrane potential of the heart cells and protects against resultant apoptosis.

In conclusion, the results presented in this study, showed for the first time, that the co-administrative use of Pin prevents Dox-induced cardiotoxicity by improving the antioxidant enzyme activity and mitochondrial bioenergetics of the H9c2 cells. Pin further reduced Dox-mediated apoptosis which is a key aspect in the prevention of cardiovascular dysfunction (**Figure 8**). In addition, our findings indicate that while Pin does in fact have cardioprotective properties *in vitro* (**Figure 8**), its therapeutic effect appear to not have a limiting effect on the chemotherapeutic potential of Dox when tested on MCF-7 cells. Though the findings obtained in the current study are very encouraging, further investigation using *in vivo* models are required to confirm the prophylactic effect of Pin against Dox-induced cardiotoxicity.

## Data Availability Statement

All datasets generated for this study are included in the article/supplementary material.

## Author Contributions

NS conducted all the experimental work, with some assistance from MM and SR. NS wrote and designed the manuscript. NS and RJ equally contributed to the conceptualization of the manuscript. RJ, NS, MM, LM, DV, SR, and BH approved the final draft of the manuscript.

## Funding

The work reported herein was made possible through funding by the South African Medical Research Council (SAMRC) through its Division of Research Capacity Development under the Internship Scholarship Programme. The content hereof is the sole responsibility of the authors and does not necessarily represent the official views of the SAMRC. The authors would also like to acknowledge the financial support from the SAMRC, Biomedical Research and Innovation Platform (baseline funding) through funding received from the South African National Treasury. Lastly, we would also like to acknowledge the National Research Foundation for the financial support [Thuthuka Grant (UID107261)]. The authors would also like to thank BioPharm, New Zealand, for supplying the treatment (pinocembrin).

## Conflict of Interest

RB was employed by BioPharm Limited, NZ.

The remaining authors declare that the research was conducted in the absence of any commercial or financial relationships that could be construed as a potential conflict of interest.
